# Screening for sickle cell disease by point-of-care tests in Italy: pilot study on 1000 at risk children

**DOI:** 10.1007/s00431-025-05988-y

**Published:** 2025-01-28

**Authors:** Maddalena Casale, Saverio Scianguetta, Teresa Palma, Laura Pinfildi, Giampiero Vallefuoco, Maria Chiara Capellupo, Domenico Roberti, Silverio Perrotta

**Affiliations:** Department of Woman, Child and General and Specialized Surgery, University of Campania ‘Luigi Vanvitelli’, Via Luigi De Crecchio 4, Naples, Italy

**Keywords:** Care access, Diagnosis, Point-of-care tests, Screening, Sickle cell

## Abstract

Sickle cell disease (SCD) is a global health problem causing premature deaths and preventable severe chronic complications. A priority goal to improve outcomes both in the short and long term is the screening for early diagnosis and access to specialized care. In Italy, as in other countries, no systematic national screening program is available. A regional pilot project was developed with the aim to screen 1000 children at risk of SCD in Italy. Primary care paediatricians received point-of-care tests (POCTs) to detect abnormal haemoglobin (Hb) to be offered to children regularly followed at their own clinics. Children positive to the POCT were referred to the regional paediatric specialized centre for diagnosis confirmation and follow up. Among 1000 at risk children screened, 85 (8,5%) tested positive for an abnormal Hb. HbS trait was reported in 69 (7%) children, HbC trait in 13 (1,3%) and SCD was diagnosed in 3 (0,3% overall; 0,56% in African background) children. African family background was the most affected by sickle mutations and all children with SCD had African ancestry. Only 56/259 (22%) primary care paediatricians invited but 20/21 (95%) reception centres adhered to the pilot screening project.

*Conclusions*: A screening program for SCD performed by the primary care paediatricians is feasible and relatively easy to organize. SCD affects mainly children with African family background and the scarce adherence of primary care paediatricians, in contrast to the high adhesion of charitable institutions, outlines the need for a mandatory screening for SCD, and improved awareness among health care providers.

**Table:** 

**What is Known:** • *Sickle Cell Disease (SCD) is a serious global health problem that requires management in specialized centres from the first months of life*. • *In the absence of neonatal screening for SCD, primary health care settings represent a feasible and costeffective approach for early disease detection*.
**What is New:** • *In Italy, screening for SCD performed by primary care pediatricians detected a hemoglobin variant in 8.5% children, with a disease prevalence of 0.3% in the whole population and 0.56% in children with African family background*. • *The poor adherence of paediatricians to the voluntary screening for SCD highlights the need for legislative interventions and training activities to ensure early diagnosis and rapid access to care for all children affected by SCD*.

**What is Known:**

• *Sickle Cell Disease (SCD) is a serious global health problem that requires management in specialized centres from the first months of life*.

• *In the absence of neonatal screening for SCD, primary health care settings represent a feasible and costeffective approach for early disease detection*.

**What is New:**

• *In Italy, screening for SCD performed by primary care pediatricians detected a hemoglobin variant in 8.5% children, with a disease prevalence of 0.3% in the whole population and 0.56% in children with African family background*.

• *The poor adherence of paediatricians to the voluntary screening for SCD highlights the need for legislative interventions and training activities to ensure early diagnosis and rapid access to care for all children affected by SCD*.

## Introduction

Sickle cell disease (SCD) is a severe congenital haemolytic anaemia due to beta globin genes’ variants resulting in relative or absolute predominance of an abnormal haemoglobin (HbS) associated or not with other haemoglobin defects (HbS/betaThal, HbS/C, HbS/D, etc.). Due to the HbS tendency for polymerisation in deoxygenated erythrocytes, severe vaso-occlusive events occur in affected individuals, injuring almost all organs (brain, spleen, lungs, bones, kidneys, etc.) with progressive acute and chronic damage, reduced survival and quality of life and high healthcare costs [1].

SCD is a non-communicable disease that causes 50–90% under-five mortality in low- and medium-resource countries [2]. In high-resource countries, such as Italy, rapid and early access to care within an efficient national healthcare system have significantly improved mortality. However, an increase in emergency room and hospital admissions for serious acute events in undiagnosed individuals with SCD has been observed [3–6]. These observations have also been reported in other countries, especially in the absence of a universal and organised screening program for the disease [2].

The geographical boundaries of the disease, traditionally limited in the African, Indian, South American, Middle East areas and Mediterranean basin, have expanded considerably, involving virtually all countries of the world, where until recently the prevalence of SCD was very low. This enormous epidemiological change of the disease has led the two major global health institutions, the World Health Organisation and the United Nations, to define SCD as an emerging global health problem and to identify screening program as an urgent health care priority [7, 8].

Some countries have already activated targeted or universal neonatal screening for SCD (USA, UK, France) but most countries in the world have not yet implemented interventions for early diagnosis of the disease.

The Italian law regulating neonatal screening currently excludes haemoglobinopathies from the list of diseases to be included in expanded neonatal screening. According to the Italian National Institute of Statistics (ISTAT), there are over 5 million individuals of foreign birth officially registered as resident in Italy (i.e. 8.9% of the total resident population) and at least 50% of the foreigners living in Southern Italy come from regions with a high prevalence of HbS carriers (https://demo.istat.it/?l=en ; last access: 27/12/2024).

Therefore, in the absence of a national screening regulated by law, a few Italian centres have organised pilot projects centre-based [9], or province-based [10], all focusing on early diagnosis of SCD in the neonatal age.

In contrast to some metabolic diseases or congenital hypothyroidism, diagnosis of SCD is not strictly required in the neonatal period but must be made within the first few months of life, to start antibiotic prophylaxis, specific treatment such as hydroxycarbamide, and to surveil for the onset of severe complications, such as cerebral stroke [11–13], thus allowing a unique screening paradigm.

The availability of point-of-care tests (POCT) for the rapid diagnosis of haemoglobin variants has paved the way for an innovative, cost-effective and rapid approach for the early diagnosis of SCD [11]. Several experiences with the use of POCT in primary health care settings, such as immunization clinics, showed the feasibility of a SCD screening program performed concurrently to established health care visits within the first months of age in low-resource countries [14].

We designed a pilot regional programme of screening for SCD with POCT at primary care paediatricians, with the aim of testing 1000 children at risk of SCD in Italy.

## Methods

Primary care paediatricians in the Campania Region (Southern Italy) were contacted by the coordinating centre (Paediatric Haematology and Oncology Centre, University Hospital Luigi Vanvitelli, Naples) through the regional scientific societies, medical associations, paediatric unions. Project and procedures were presented through specific documentation, webinars and informative meetings.

Primary care paediatricians adhering to the project were asked to fill in a dedicated form reporting the number of children at high risk for SCD among those children assisted in their own clinics. High-risk children for SCD were defined as male and female children from 1 month to 18 years of age, with at least one parent from: Africa, India, Central and South America, Middle East, Greece, Albania and Sicily.

As in Italy, many at risk children are migrants or refugees, reception centres accommodating children at risk were contacted and involved in the project.

Once the completed form had been received, the coordinating centre prepared study material in accordance with the number of children selected by the primary care paediatrician (informative documentation, informed consent, material for carrying out the rapid tests) and delivered it to the office of primary care paediatricians, who offered and carried out the rapid tests to selected children, during a visit or a clinical practice check-up, after signing the informed consent. Paediatricians who responded to the questionnaire, after assessment of their patients, were considered as responding, even if they did not identify children defined at risk per protocol.

The paediatricians filled in a form containing demographic data and the result of the rapid tests. All children with a haemoglobin variant detected on the rapid test were referred to the project coordinating centre for diagnostic confirmation with high-performance liquid chromatography (HPLC) and molecular diagnosis, as per established clinical practice [15].

For this project, the Sickle SCAN® (BioMedomics Inc., Morrisville, NC, USA) and HemoTypeSC ™ (Silver Lake Research, Azusa, CA, USA) were used as POCT.

The research protocol was approved by the local ethical committees Università degli Studi della Campania “Luigi Vanvitelli” – Azienda Ospedaliera Universitaria “Luigi Vanvitelli”—AORN “Ospedali dei Colli” no. 0024026/i del 08/08/2021 and no. 0035151/i del 09/12/2021 and was implemented in accordance with the Declaration of Helsinki and the ICH guidelines for good clinical practice. All parents and patients received verbal and written explanation of the aims and procedure of the study and written informed consent was obtained.

### Statistical analysis

IBM SPSS Statistics software (version 28) was used for the statistical analysis. An initial descriptive statistical analysis of the variables was performed. Quantitative variables were analyzed using central tendency and variability indexes; categorical variables were analyzed according to frequency and percentage (*n*, %). Children tested positive at POCT underwent a descriptive statistical analysis.

## Results

**Fig. 1:**
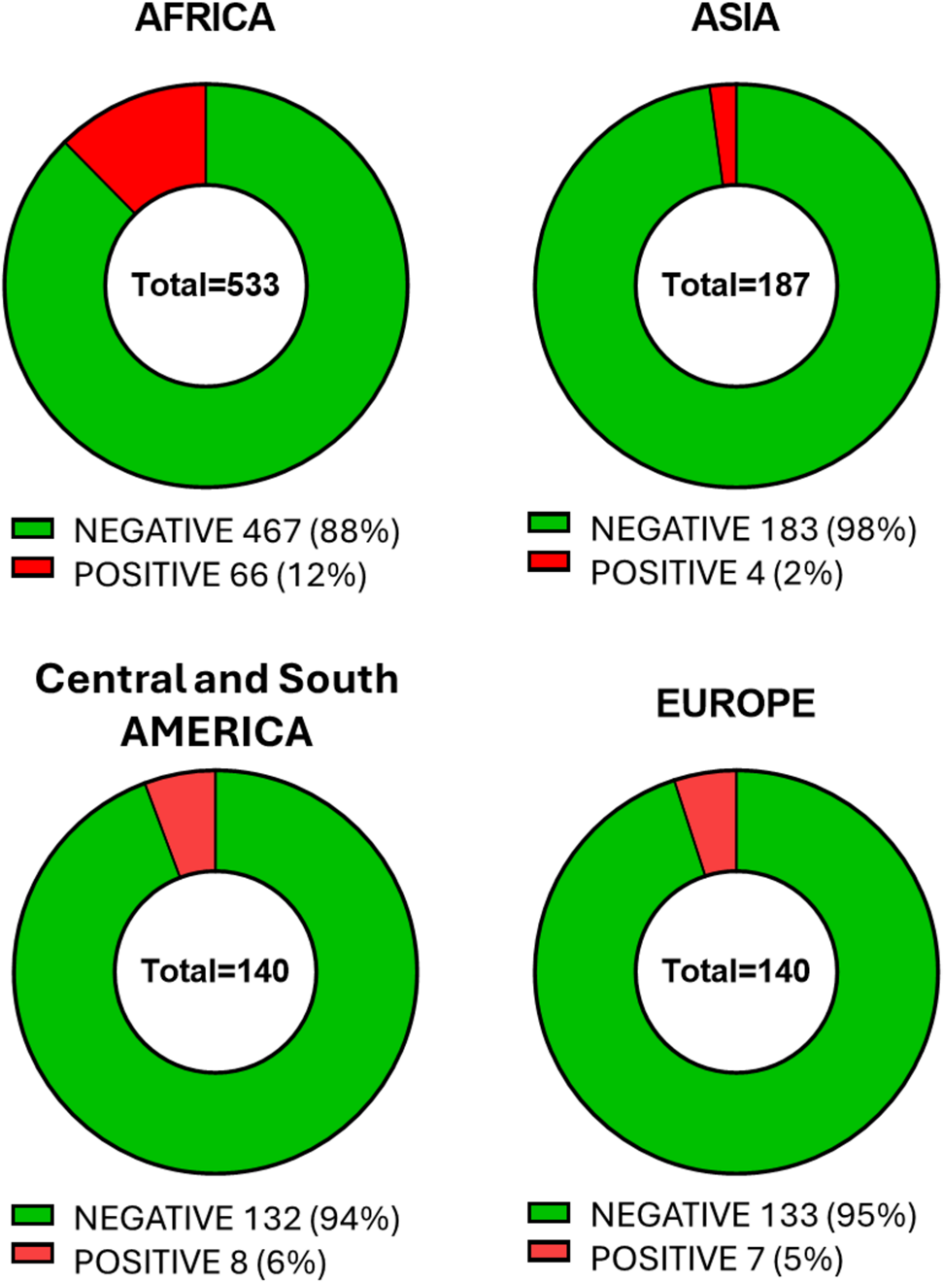
SCD screening results according to different family background

One thousand children, 381 females and 619 males, were screened at an average age of 8.5 years (SD 6.5) and 85 children (8.5%) tested positive (31 females and 54 males) (Table 1). Although the highest rate of positive tests was observed in African family background, the positivity rate in children with the other geographical ancestry ranged between 2 and 7%, thus showing that the sickle mutations are widely spread globally (Fig. 1).

**Table 1:** SCD screening results on the overall population tested (1000 children)

Family background	Total	Negative	Positive
Africa	533 (53.3%)	467 (46.7%)	66 (6.6%)
Asia	187 (18.7%)	183 (18.3%)	4 (0.4%)
Europe	140 (14%)	133 (13.3%)	7 (0.7%)
Central and South America	140 (14%)	132 (13.2%)	8 (0.8%)
Total	1000 (100%)	915 (91.5%)	85 (8.5%)

**Fig. 2:**
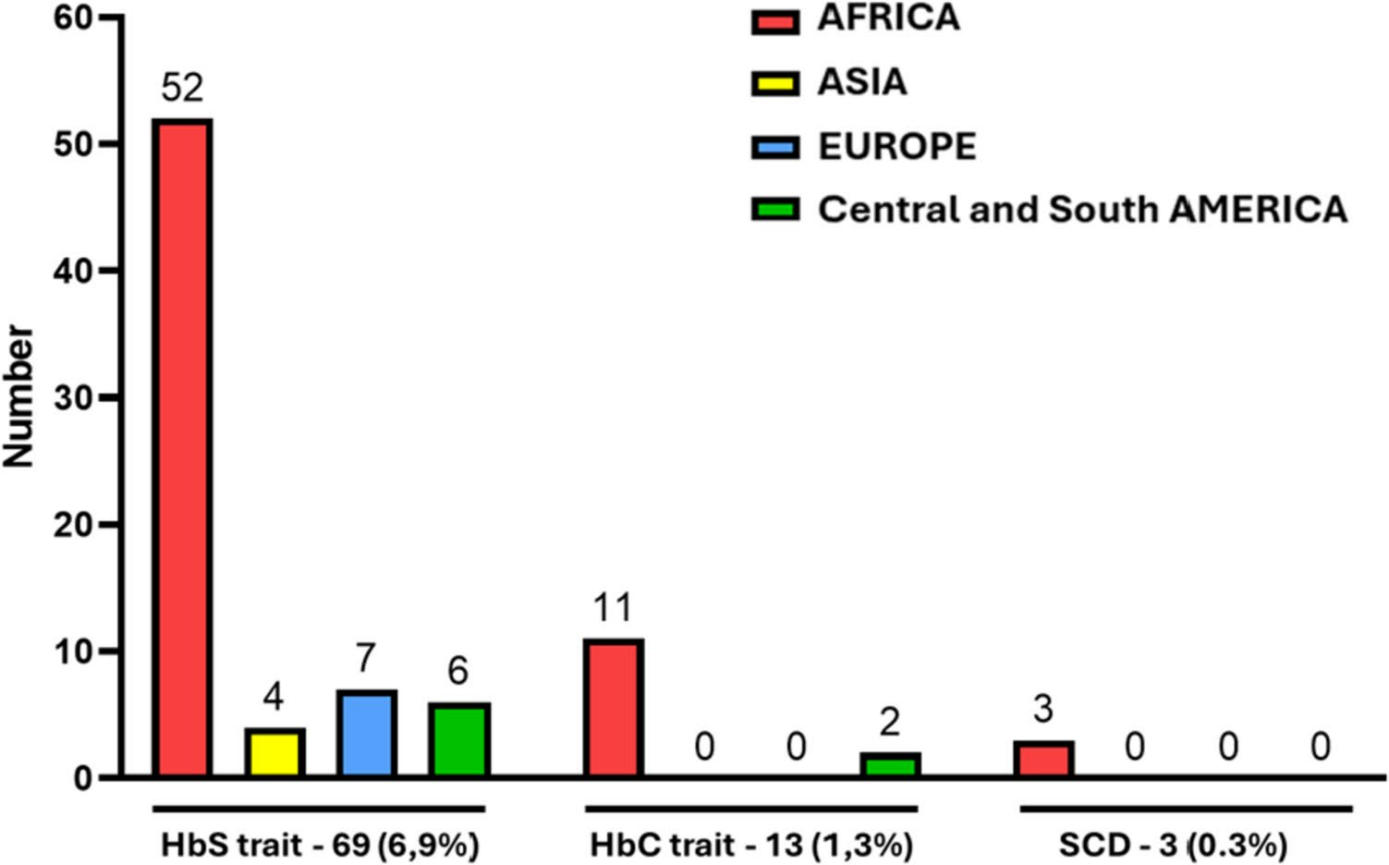
SCD screening results according to the types of mutation

HbS trait was reported in 69 (7%) children, 52 (76%) with African ancestry, 7 (10%) with European ancestry, 6 (9%) with South American ancestry and 4 (6%) with Asian ancestry (Fig. 2). HbC trait was reported 13 (1.3%) children, 11 (85%) among African family background and 2 (15%) from European family background. Three children were affected by SCD (SC) with a prevalence of 0.3% in the whole screened sample and 0.56% in the African family background.

Invalid tests were reported in 19 (2%) children (6 false positive tests and 13 tests of doubtful interpretation) due to technical failure of a batch of the POCTs (Sickle SCAN®) and required traditional screening. After having performed internal tests with known controls and having found malfunction and doubtful interpretation of the POCT results, Sickle SCAN® was no longer used in the project and replaced with other POCTs.

Among 259 paediatricians invited to participate to the pilot screening programme, only 56 (22%) adhered to the voluntary screening programme. Among 203 non-participating paediatricians, 191 (94%) did not respond to the invitation, despite several requests, 6 (3%) refused participation due to lack of time or disinterest; 6 (3%) were retired and did not provide contacts of their replacement.

**Fig. 3:**
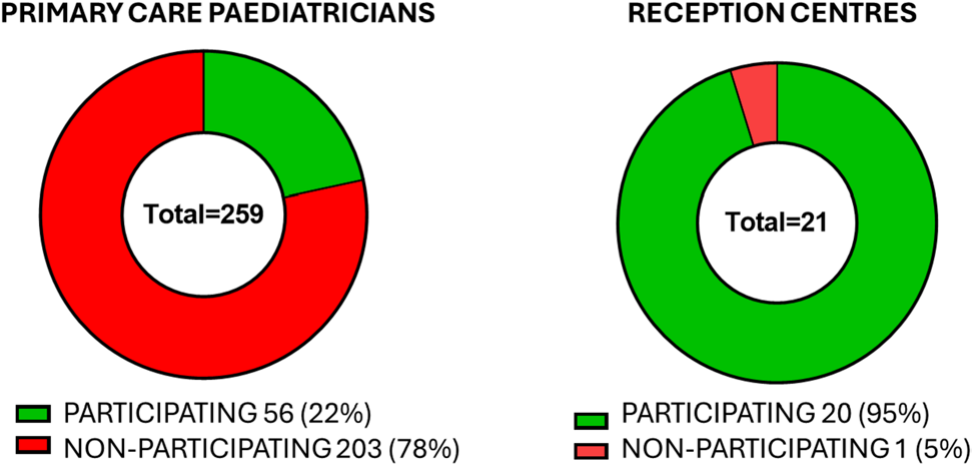
Adherence to the SCD screening among primary care paediatricians and reception centres

In contrast, among 21 reception centres contacted by the coordinating centre, 20 (95%) adhered to the project and organized internal screening, involving primary care paediatricians for the diagnostic confirmation (Fig. 3).

## Discussion

SCD causes a high health and social burden, as it is the world-wide most frequent genetic disease, affecting more than 300,000 children in 2010 with a calculated projected growth rate of 32.5% in 2050 [16]. It is highly prevalent among individuals with African background, who often suffer from limitations related to language barriers, restricted social and economic integration in the countries of arrival, thus reducing or delaying the access to early diagnosis and appropriate medical care [4, 17].

However, SCD-related acute and chronic complications can be prevented or timely and rightly managed through early diagnosis [2].

All the aforementioned factors clarify why it is crucial to organise screening programmes, as recommended by the major health institutions. Some high-resource countries have organised neonatal screenings with differences in regard to population (targeted or universal) and extent of the programme (national, regional, centre-based) [2].

Italy, like many other countries in the world, has not implemented a national neonatal screening for hemoglobinopathies due to limitations of current national legislation and differences in regional health policies [18]. The number of carriers of Hb variants in our study, as high as 8.5%, is in line with the general prevalence of Hb disorders reported in 7% of the population, considering that our study targeted high risk population due to geographical origin. However, it is very difficult to compare the positivity rates for Hb variants in different studies, given the different target population involved (universal screening, targeted screening, population at highest risk) [9, 10].

Universal neonatal screening is certainly the most systematic and coordinated way to implement a programme of early diagnosis and care, but the costs, complex logistical procedures and the need for an efficient coordination system limit the implementation of a nationwide and lasting neonatal screening programme even in countries with high economic resources [2], with the final results of inequity in care access for affected children. In addition, neonatal persistence of fetal Hb, the most potent known inhibitor of HbS polymerisation, allows to start the regular follow up and preventative measures (antibiotic prophylaxis, vaccinations, monitoring of complications and hydroxycarbamide) within the first months of life and not necessarily in the neonatal period [11]. This allows unique and creative screening strategies that best suit the social, economic and legislative context of the different countries or regions, with the ultimate goal of screening all children within the first months of age [2].

The Italian National Health System provides comprehensive health care covering the entire population, irrespective of income or contributions, employment, or preexisting health conditions. The whole country is divided into several health districts, most of them are covered by primary care paediatricians who provide general first-access care for all children and adolescents (0–16 years) free of charge. Furthermore, Italian Ministry of Health charged primary care paediatricians to perform preventative medicine and care through routine checkup examinations regularly scheduled, following national and regional programs, which are designed in collaboration with the Ministry of Health, and with the regional governments at the local level [19].

For all these reasons, a screening programme carried out by physicians working for the National Health System could be a feasible approach throughout the entire country, overcoming the major barriers that have so far halted the implementation of a national screening programme for SCD in Italy. Moreover, in the Netherlands, where a neonatal screening programme is implemented, one-third of the newly diagnosed patients were reported in migrants born outside the country and not identified through the neonatal screening [20]. This outlines the need for complementary screening strategies to detect affected children which do not benefit from neonatal screening, thus reducing further disparities in healthcare access in a disadvantaged group, such as migrant and adopted children.

Combining screening tests with a routine checkup increases screening adherence and reduces costs for families and for the national health system. In our experience, families always accepted the screening test, when it was offered by the primary care paediatricians or by the managers of the reception centres, thus suggesting the families' trust in the reference figures providing assistance. Furthermore, the access to specialized care is improved when the screening is performed by the primary health care providers, who refer patients affected to the specialized clinical centre. This has been reported for SCD screening approach using POCT at the primary care immunization centre in low-income countries [14]. Real life studies using POCTs for detecting Hb variants on large populations reported overall diagnostic accuracy of 99% [14], with sensitivity of 100–93.4% and specificity 100–99% for HemoTypeSC™ (Silver Lake Research, Azusa, CA, USA); sensitivity 100–90% and specificity 100–92.6% for Sickle SCAN® (BioMedomics Inc., Morrisville, NC, USA). Therefore, our data are in line with published data, although we have decided to no longer use Sickle SCAN® in the project due to technical failure of a large number of tests purchased for the project.

However, the very poor adherence of primary care paediatricians to the pilot project, with rates as low as 22% of invited paediatricians, highlights a critical element: screening for SCD cannot rely on a voluntary basis, but legislative interventions are needed to ensure its implementation by all primary care paediatricians or health professionals in charge of children. The high adhesion of charitable organisations, such as Caritas, Emergency and all the associations involved in the process of accommodation for foreigners and migrants, underlines once again how SCD is one of the most neglected diseases globally and is not considered a priority for primary care paediatricians still nowadays [17, 21].

Italy recently initiated a screening program for type 1 diabetes and coeliac disease as part of the public health program provided by primary care paediatricians by law, and is indeed target of international interest.

A pilot study has been sponsored by the Italian National Institute of Health and is ongoing in several Italian regions, including Campania, to evaluate the best approach to implement screening for type 1 diabetes and celiac disease nationwide. This law was unanimously voted by the Italian Parliament thanks to the pressure of patient associations and people from the diabetes and scientific community advocating for a dedicated screening programme [22]. Unfortunately, data presented in this manuscript show once again that SCD is the most mistreated disease, also due to the absence of a strong representation of patient associations in the different countries, as SCD disproportionately affects economically disadvantaged groups suffering from health inequities and disparities [23, 24].

Our results align with the main results and conclusions from previous and ongoing pilot SCD screening programs: community involvement to advocate a dedicated screening and government involvement to integrate the SCD screening into the local health system are critical determinants for success [25].

Thus, our work showed that screening for SCD performed by primary care paediatricians or general caregivers is feasible and relatively easy to implement.

However, the screening for SCD should be incorporated into the core health system, by means of a national legislative intervention, to offer early diagnosis for all children as soon as possible and within the first months of age. In Italy, SCD could be indeed associated with the incoming screening for type 1 diabetes and coeliac disease by law. This approach can be applied to different contexts, wherever primary care service free of charge for families is available, in low and high-resource countries.

National and international charitable organisations, with the potential for a higher impact on institutions or policymakers, should identify SCD as a paediatric health priority, in low and high-income countries, and should advocate for SCD screening to become part of the global health program.

## Data Availability

The data that support the findings of this study are available from the corresponding author upon reasonable request.
